# Correlation among the Power Dissipation Efficiency, Flow Stress Course, and Activation Energy Evolution in Cr-Mo Low-Alloyed Steel

**DOI:** 10.3390/ma13163480

**Published:** 2020-08-07

**Authors:** Petr Opěla, Ivo Schindler, Petr Kawulok, Rostislav Kawulok, Stanislav Rusz, Horymír Navrátil, Radek Jurča

**Affiliations:** 1 Faculty of Materials Science and Technology, VSB–Technical University of Ostrava, 17. listopadu 2172/15, 70800 Ostrava–Poruba, Czech Republic; ivo.schindler@vsb.cz (I.S.); petr.kawulok@vsb.cz (P.K.); rostislav.kawulok@vsb.cz (R.K.); stanislav.rusz2@vsb.cz (S.R.); horymir.navratil@vsb.cz (H.N.); 2 Třinecké železárny, a.s., Průmyslová 1000, 739 61 Třinec–Staré Město, Czech Republic; radek.jurca@trz.cz

**Keywords:** processing maps, activation energy maps, flow stress maps, artificial neural networks

## Abstract

In the presented research, conventional hot processing maps superimposed over the flow stress maps or activation energy maps are utilized to study a correlation among the efficiency of power dissipation, flow stress, and activation energy evolution in the case of Cr-Mo low-alloyed steel. All maps have been assembled on the basis of two flow curve datasets. The experimental one is the result of series of uniaxial hot compression tests. The predicted one has been calculated on the basis of the subsequent approximation procedure via a well-adapted artificial neural network. It was found that both flow stress and activation energy evolution are capable of expressing changes in the studied steel caused by the hot compression deformation. A direct association with the course of power dissipation efficiency is then evident in the case of both. The connection of the presence of instability districts to the activation energy evolution, flow stress course, and power dissipation efficiency was discussed further. Based on the obtained findings it can be stated that the activation energy processing maps represent another tool for the finding of appropriate forming conditions and can be utilized as a support feature for the conventionally-used processing maps to extend their informative ability.

## 1. Introduction

Since the end of the 2nd millennium, hot processing maps, introduced on the basis of the dynamic material model (DMM), have been being broadly used in the sense of the optimization of hot forming processes (forging, rolling, etc.) [1,2,3,4,5,6,7,8,9,10,11,12,13,14,15,16,17,18,19,20,21,22,23,24,25,26,27,28]. It is well known that the processing map displays the distribution of power dissipation efficiency and metallurgical instability in the strain rate–temperature coordinates under the specific value of strain. The thermomechanical conditions linked with the higher efficiency of power dissipation and in the same time with the absence of metallurgical instability are then usually considered as advantageous. In the case of a specific material, the results are usually presented as the series of processing maps assembled at various strains or as a volumetric chart, which allows these maps to be unified into one coherent unit [1,2].

The above approach has been employed to study the hot working behavior of various materials which were prepared and formed by different methods and subsequently used for different applications—for instance, steels: Fe-11Mn-10Al-0.9C duplex low-density steel susceptible to κ-carbides [3], as-extruded 42CrMo high-strength steel [4], 25Cr3Mo3NiNb steel [5], 10CrMo9-10 steel [6], 34CrNiMo medium carbon steel [7], medium carbon steel, microalloyed by B and Ti [8], medium-carbon bainitic steel [9], 43CrNi steel [10], high-carbon/low-carbon steel composite [11], 347H austenitic heat-resistant stainless steel [12], high-titanium Nb-micro-alloyed steel [2]; nickel alloys: Ni-based superalloy [13], P/M nickel-based superalloy [14], IN-718 superalloy [15], NiTiNb shape memory alloy [16]; aluminum alloys: as-extruded 7075 [17], 5052 alloy [1], B_4_C/6061Al nanocomposites fabricated by spark plasma sintering [18]; titanium alloys: Ti-15-3 [19], Ti-6242 [20], ATI425 [21], TC21 [22]; zirconium alloys: reactor-grade alpha-zirconium [23], Zr-2.5Nb [24]; and some other alloys: AZ31-1Ca-1.5 vol% nano-alumina composite [25], Cu–Cr–Zr–Nd alloy [26], Pb-Mg-10Al-0.5B alloy [27], Fe_3_Al intermetallic alloy [28], etc.

Recently, some efforts have been made to improve the informative capability of the above-mentioned processing maps. In the case of a specific material, these maps are usually developed on the basis of an experimentally-achieved flow-curve dataset. Nevertheless, Quan et al. [17] have proposed to extend an experimental dataset by means of a prediction procedure since the limited number of experimental curves can lead to the inferior informative ability of compiled processing maps. This suggestion has been recently studied in the case of 10CrMo9-10 steel [6] when the additional (predicted) dataset revealed potentially inappropriate forming conditions. In connection with the above presented maps, Zhou et al. [1] utilized a term—conventional hot processing (CHP) maps. They pointed out the fact that, until now, employed processing maps do not take into account the difficulty of the course of material deformation. They demonstrated this assumption on the previously-published CHP maps—these maps indicated suitable forming conditions at thermomechanical circumstances in which the corresponding microstructure observations revealed notable shear bands [1]. Zhou et al. therefore introduced a so-called activation energy processing (AEP) maps. These maps combine the above discussed CHP maps and activation-energy (AE) maps. The activation energy characterizes the difficulty of a deformation course since embodies the capability of atoms to surmount energy barriers. Lower values of activation energy are then linked with the easier course of deformation. Thus, the potentially appropriate forming conditions are in the case of the AEP maps given by the high values of the efficiency of power dissipation in combination with the absence of instability regions and in the same time by the low values of activation energy [1,8,14,29]. In the case of the 5052-aluminum alloy, Zhou et al. [1] have confirmed that the introduced AEP maps display the suitable and aggravated forming conditions in comparison with the CHP maps more accurately.

The aim of the presented research is to find a correlation between the efficiency of power dissipation and the evolution of the activation energy for the case of Cr-Mo low-alloyed steel, and, in addition, also consider the relation of power dissipation efficiency to the flow stress course. This means the assembly of the above-mentioned AEP maps (i.e., the combination of CHP and AE maps) and creating the combination of the CHP maps with flow-stress (FS) maps will be realized. In addition, the relation of the presence of instability districts in the assembled CHP maps to the AEP maps and FS maps will be also taken into account.

## 2. Materials and Methods

### 2.1. Experimental Procedure

The chemical composition of the investigated Cr-Mo low-alloyed steel is displayed in Table 1. Cylindrical hot-compression-test samples with the diameter of 10 mm and the length of 15 mm (prepared by turning) were subjected to the series of uniaxial hot compression tests on the Gleeble 3800 in connection with the Hydrawedge II Mobile Conversion Unit (Dynamic Systems Inc., Poestenkill, NY, USA) [30].

Four strain rates (specifically: 0.02, 0.2, 2, and 20 s^−1^) in combination with six deformation temperatures (1043, 1113, 1203, 1303, 1413, and 1553 K) have been tested in the frame of the current research, when the value of true strain reached to 1.0. Before the compression, each tested specimen was preheated to a deformation temperature by the heating rate of 5 K·s^−1^ (performed via direct electric resistance heating) with the following dwell time of 300 s. The temperature measurement was always realized on the sample surface in the middle length. This measurement was mediated via a pair of thermocouple wires (fixed by welding) of the K-type (i.e., Ni–Cr (+) and Ni–Al (−)) and R-type (Pt–13%Rh (+) and Pt (−)) as regards to the temperatures of 1043 K–1413 K and the temperature of 1553 K, respectively. The testing course was always performed under vacuum in order to prohibit oxidation processes. Tantalum foils in combination with a nickel-based high-temperature grease were chosen to protect the anvils and reduce the friction on the sample-anvils interface. The described experimental procedure resulted in a flow curve dataset expressing the evolution of true flow stress under the above-mentioned experimental values of true strain, strain rate and deformation temperature.

After the prime evaluation of subsequently assembled CHP-FS and AEP maps, additional compression tests were performed under various thermomechanical conditions in order to link these maps with a metallographic observation. The compressed specimens were immediately water-quenched to fix the structure, thereafter sectioned along the compression axis, mechanically polished, and then etched (solution of picric acid (50 g) and ferric chloride (5 g) in 100 mL of distilled water) to visualize the original grain boundaries. An optical microscopy (OM) observation has been subsequently realized by means of the Olympus GX51 inverted metallurgical microscope (Olympus Corporation, Tokyo, Japan).

### 2.2. Flow Curve Approximation and Prediction

Since the experimentally obtained flow curve dataset is intended to be used for the creation of CHP and AEP maps which is associated with the interpolation of the processed data, the approximation and subsequent prediction processes were applied to augment the input dataset and, thus, increase the interpolation accuracy.

**Figure 1:**
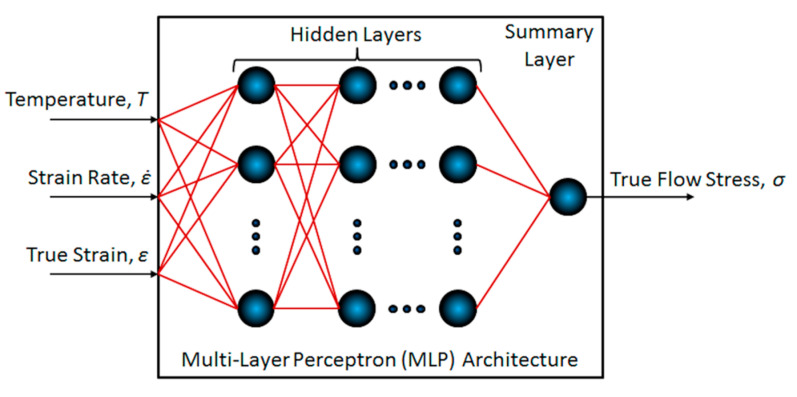
General architecture of the employed multi-layer perceptron (MLP) network.

Based on the previous experiences [6,31,32,33] and other various studies that have been done in recent decades (see, e.g., introduction in [31]), the artificial neural network (ANN) approach [34,35,36] was found to be appropriate to deal with the current approximation and prediction task. Specifically, a multi-layer perceptron (MLP) network type with the feed-forward course has been employed in order to create a functional relationship between the vectors of the independent variables (i.e., true strain, *ε* (-), strain rate $\dot{\varepsilon }$ (s^−1^), and temperature, *T* (K)) and the vector of the dependent variable (i.e., true flow stress, *σ* (MPa). Generally, the functional relationship (MLP architecture) is given by the ad hoc established set of perceptrons (computational units) [35] which are arranged into one or more hidden layers and one summary layer, when the perceptrons of adjacent layers are connected via synaptic weights (material constants), see Figure 1. The detailed description of the ANN approximation background inclusive of MLP network type can be found, e.g., in [36].

Based on the adaptation procedure (analogical to that utilized in [6]), an ideal MLP-network architecture was found to be given by two hidden layers, each with eight perceptrons; these hidden perceptrons were activated via a hyperbolic-tangent sigmoid activation function [37].

Synaptic weights (material constants) were calculated on the basis of the minimization of the mean squared error [38] of MLP-output residuals (learning procedure). This minimization task has been solved by the use of the Levenberg–Marquardt algorithm [39,40,41] in a combination with the Bayesian regularization [42,43] and the back-propagation of error signal [44]. Note, 12 experimental flow curves were subjected to the minimization algorithm (training set), while six curves were used to evaluate the prediction ability during the training course (validation set) and other six curves then served for the subsequent final prediction evaluation of a trained MLP architecture (testing set)—see Table 2.

It should be noted that the input vectors ($\varepsilon ,\;\dot{\varepsilon },\;T$) are introduced to the network in a normalized form (i.e., dimensionless and with the sample standard deviations equal to 1.0). The applied normalization procedure was analogical to that described previously in [6].

Based on the assembled MLP network, flow curve prediction has been realized for five additional temperatures (1078, 1158, 1253, 1358, and 1483 K) under the experimentally tested strain rate levels.

The entire approximation and prediction procedures have been performed by means of MATLAB^®^ 9.3 software [45] with the embedded Neural Network Toolbox™ 11.0 (MathWorks^®^, Natick, MA, USA) [46].

### 2.3. Conventional Hot Processing Maps

In the current research, CHP maps of the studied steel were compiled on the basis of the findings resulting from the Prasad’s dynamic material model (DMM) [20,47,48]. With regard to the hot-formed workpiece, the DMM discloses a related energy-balance background. Specifically, it deals with the dissipation of power in the connection with a plastic deformation and associated metallurgical processes like, e.g., dynamic recovery (DRV) and recrystallization (DRX). On the foreground of the DMM theory, a dimensionless indicator is utilized to quantify the power dissipation in the wide range of thermomechanical circumstances, just with the relation to the microstructural changes—known as the efficiency of power dissipation, *η* (-, %) [20]:

(1) $$\eta =\frac{2\cdot m}{m+1},$$

where the strain rate sensitivity, *m* (-), is given as [20]:

(2) $$m={\frac{\partial \ln\sigma }{\partial \ln\dot{\varepsilon }}|}_{T,\varepsilon },$$

Note that in order to perform this derivation, the relevant ln *σ*—$\ln\dot{\varepsilon }$ data points were extracted from the combined experimental/predicted flow curve dataset.

The dependency of the efficiency of power dissipation with respect to the temperature and strain rate is under specific strains usually graphically expressed in the form of so-called power dissipation maps. These maps are usually combined with the so-called flow instability maps, when the superimposition of both map types resulting in the above introduced CHP maps. The presence of instability is conditioned by the continuum criterion [49,50]:

(3) $$\xi \left(\dot{\varepsilon }\right)=\frac{\partial \ln\left(\frac{m}{m+1}\right)}{\partial \ln\dot{\varepsilon }}+m\leq 0,$$

where the value of *ξ*($\dot{\varepsilon }$) (-) is known as the flow instability parameter.

### 2.4. Activation-Energy Maps

As it was mentioned in the introduction, the AEP maps, i.e., the combination of the CHP maps and the activation energy (AE) maps, can enhance the ability of CHP maps to reveal the potentially unstable forming conditions since the activation energy has the ability to reflect the difficulty of deformation course [1]. The creation of the AE maps requires the calculation of activation energy values under all thermomechanical conditions. In most cases, however, the activation energy is considered to be a material constant (so-called apparent activation energy) or, at most, strain dependent [21,26,51,52,53,54]. In order to deal with the activation energy as a strain, strain rate, and temperature dependent parameter, the method utilized, e.g., in [55,56] has been employed. Similarly, as in the case of the apparent activation energy, the calculation is based on the well-known Garofalo’s relationship [57]. With the use of this relation, the values of the activation energy, *Q* ($\varepsilon ,\;\dot{\varepsilon },\;T$) (J·mol^−1^), have been calculated as follows [55,56]:

(4) $$Q\left(\varepsilon ,\dot{\varepsilon },T\right)=R\cdot n\left(\varepsilon ,T\right)\cdot M\left(\varepsilon ,\dot{\varepsilon }\right),$$

where the *R* (8.314 J·K^−1^·mol^−1^) is the universal gas constant, and the products of the *n*(*ε*,*T*) (-) and *M*(*ε*,$\;\dot{\varepsilon }$) (K) parameters are given on the basis of the known values (experimental and predicted via MLP) of $\dot{\varepsilon }$*, T* and *σ* as follows [55,56]:

(5) $$n\left(\varepsilon ,T\right)\cdot M\left(\varepsilon ,\dot{\varepsilon }\right)={\frac{\partial \ln\dot{\varepsilon }}{\partial \ln\left\{\sinh\left[\alpha \left(\varepsilon ,T\right)\cdot \sigma \right]\right\}}|}_{\varepsilon ,T}\cdot {\frac{\partial \ln\left\{\sinh\left[\alpha \left(\varepsilon \right)\cdot \sigma \right]\right\}}{\partial \left(1/T\right)}|}_{\varepsilon ,\dot{\varepsilon }},$$

where the values of the stress multiplier *α*(*ε*,*T*) (MPa^−1^) were estimated as [55,56]:

(6) $$\alpha \left(\varepsilon ,T\right)={\frac{\partial \ln\dot{\varepsilon }}{\partial \sigma }|}_{\varepsilon ,T}/{\frac{\partial \ln\dot{\varepsilon }}{\partial \ln\sigma }|}_{\varepsilon ,T}.$$

Note that *α*(*ε*) is the arithmetic mean [58] of the *α*(*ε*,*T*) parameter under different temperatures. From the practical reasons, the above introduced parameters have been expressed in the form of the following multivariate polynomials:

(7) $$M\left(\varepsilon ,\dot{\varepsilon }\right)=\sum_{i=0}^{4}\sum_{j=0}^{4}a_{ij}\cdot \varepsilon^{i}\cdot \ln^{j}\dot{\varepsilon },$$

(8) $$n\left(\varepsilon ,T\right)=\sum_{i=0}^{4}\sum_{j=0}^{4}b_{ij}\cdot \varepsilon^{i}\cdot T^{j},$$

(9) $$\alpha \left(\varepsilon ,T\right)=\sum_{i=0}^{4}\sum_{j=0}^{4}c_{ij}\cdot \varepsilon^{i}\cdot T^{j}.$$

The material constants of the polynomials (7)–(9), i.e., *a_ij_* (-), *b_ij_* (-) and *c_ij_* (-) (where *i* = [0, 4] ⊂ ${\mathbb{N}}_{0}$ and *j* = [0, 4] ⊂ ${\mathbb{N}}_{0}$), have been estimated on the basis of nonlinear least square method via the Levenberg–Marquardt iterative optimization algorithm [39,40,41].

## 3. Results and Discussion

### 3.1. Evaluation of the Performed Calculations

In order to evaluate the accuracy of the above performed MLP-approximation, the Pearson’s correlation coefficient [59], *R* (-), and the average absolute relative error, *AARE* (%), have been calculated—see Equations (10) and (11) [17]. In these equations, the *T_i_* (MPa) and *A_i_* (MPa) represent the flow stress values of the target (i.e., experimental) and approximated dataset, respectively. *i* = [1, *n*] ⊂ ℕ, where *n* is the number of elements in the flow stress vector. $\bar{T}$ (MPa) and $\bar{A}$ (MPa) then embody the mean values [58] of these vectors.

(10) $$R=\frac{\sum_{i=1}^{n}\left(T_{i}-\bar{T}\right)\cdot \left(A_{i}-\bar{A}\right)}{\sqrt{\sum_{i=1}^{n}{\left(T_{i}-\bar{T}\right)}^{2}\cdot \sum_{i=1}^{n}{\left(A_{i}-\bar{A}\right)}^{2}}},$$

(11) $$AARE=\frac{1}{n}\cdot \sum_{i=1}^{n}\left|\frac{T_{i}-A_{i}}{T_{i}}\right|\cdot 100.$$

**Figure 2:**
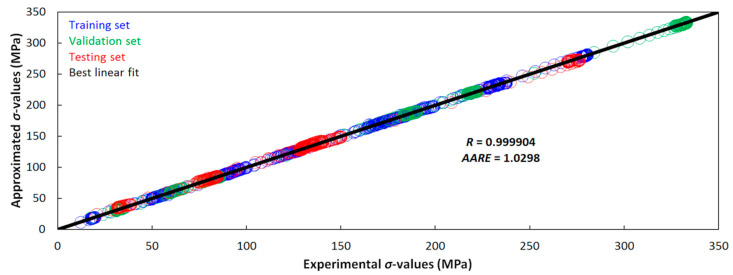
Correlation between the experimental and by-MLP approximated flow curve datasets.

As can be seen in Figure 2, both statistical indicators exhibit the favorable values. It is apparent, the approximated dataset, thanks to the utilized MLP approach, exhibits a good fit with the experimental one.

**Figure 3:**
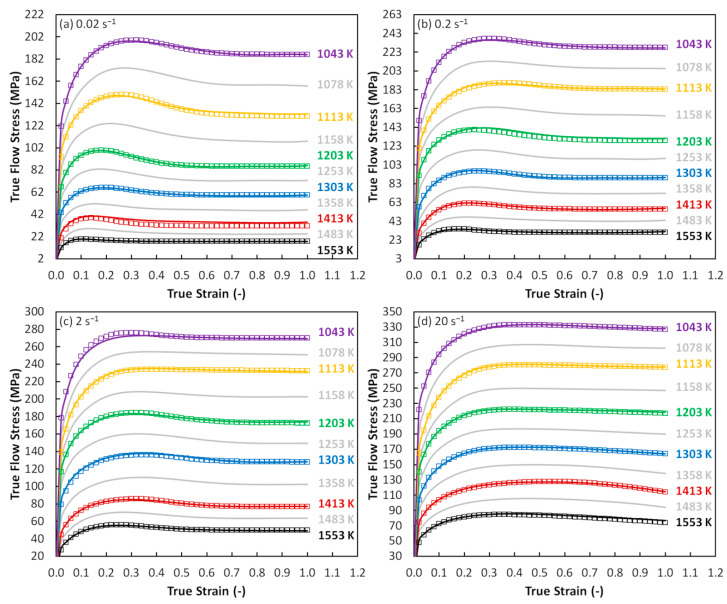
Experimental, approximated, and predicted flow curves of the examined Cr-Mo low-alloyed steel. (**a**) Strain rate of 0.02 s^−1^; (**b**) strain rate of 0.2 s^−1^; (**c**) strain rate of 2 s^−1^; (**d**) strain rate of 20 s^−1^. Boxes–experiment; color solid lines–approximation; gray solid lines–prediction.

The experimental and approximated flow curves are displayed in Figure 3 (see the color curves). In addition, the gray curves then represent the performed prediction. It can be seen, this graphical comparison confirms the above achieved statistical observation. It is also noticeable that the predicted curves fit into the presumed flow stress levels. The presented flow curves show the apparent manifestation of a DRX course and it is favorable that this characteristic flow stress course could be modeled via the assembled MLP network.

The calculated material constants of the above-introduced polynomials (7)–(9) are listed in Table 3, Table 4 and Table 5 together with the achieved values of the Pearson’s correlation coefficient [59], *R* (-), Equation (10). In this equation, in association with the polynomials (7)–(9), *T_i_* (K, -, MPa^−1^) and *A_i_* (K, -, MPa^−1^) represent the target and approximated values of the studied parameters, i.e., *M*($\varepsilon ,\;\dot{\varepsilon }$), *n*(ε, T), and *α*(*ε*,*T*). *i* = [1, *n*] ⊂ ℕ, where, *n* is the number of elements in the individual parameter vector. $\bar{T}$ (K, -, MPa^−1^) and $\bar{A}$ (K, -, MPa^−1^) then embody the mean values [58] of these vectors.

### 3.2. Flow Stress Evolution

**Figure 4:**
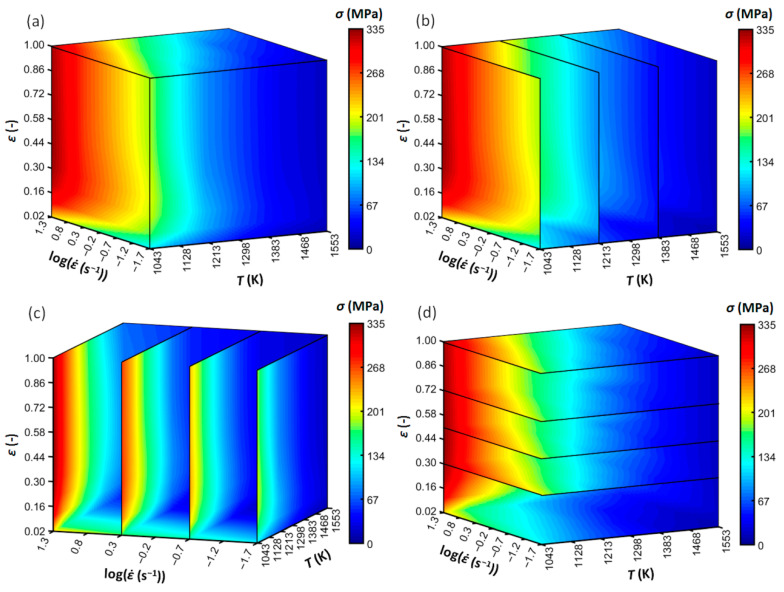
Volumetric expression of the experimental-predicted flow curve datasets of the Cr-Mo low-alloyed steel. Colors–flow-stress level. (**a**) Global overview; (**b**) sliced in the temperature axis; (**c**) sliced in the strain rate axis; (**d**) sliced in the strain axis.

The experimental and predicted flow curve datasets are expressed together in the form of a volumetric chart (constructed using MATLAB^®^ 9.3 software (MathWorks^®^, Natick, MA, USA) [45]), see Figure 4.

The independent variables, i.e., deformation temperature, strain rate, and true strain are represented by the x, y, and z axes, respectively. The flow stress values are then embodied by the 3D-color-space matrix. For a better orientation, the volumetric chart is expressed also in the form of sliced panels along the x, y, and z exes. Predictably, the flow stress level declines with an increasing deformation temperature and decreasing strain rate. A DRX-like behavior can be observed—with the increase of strain, the flow stress increases up to a maximum level (strengthening phase) and then decreases to a steady-state flow (softening phase). In the case of the highest strain rate level (20 s^−1^), however, the studied strain range is not enough to undergo through the entire DRX softening (i.e., reaching the steady-state).

### 3.3. Processing Maps

**Figure 5:**
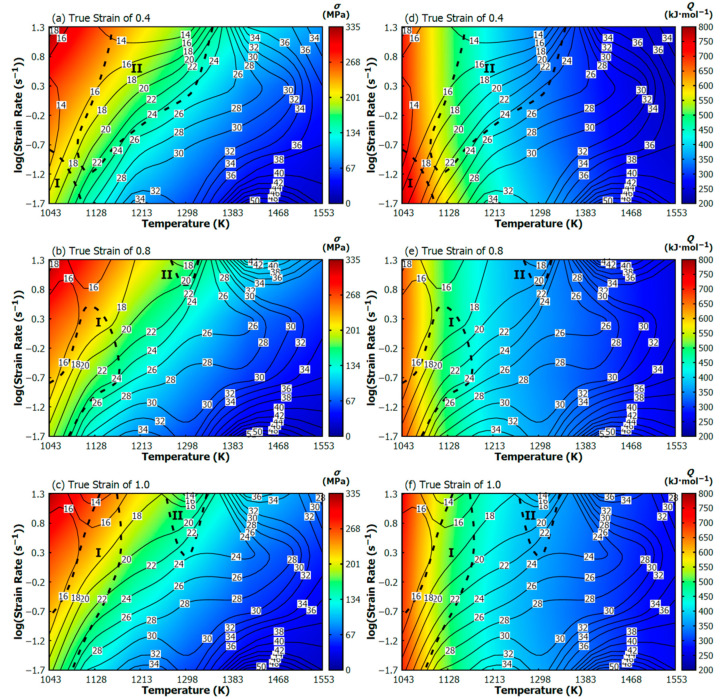
Processing maps of the investigated Cr-Mo low alloyed steel. (**a**–**c**) Conventional hot processing (CHP) maps superimposed by flow-stress (FS) maps; (**d**–**f**) activation-energy processing (AEP) maps. Solid contours with labels—power dissipation efficiency, dashed lines—districts of metallurgical instability, color background—flow stress evolution in (**a**–**c**) or activation energy evolution in (**d**–**f**).

Based on the experimental and predicted flow curve datasets (see Figure 4), the calculations introduced in Section 2.3 enabled a gain in the *η* and *ξ* values of the investigated Cr-Mo steel under the wide range of thermomechanical conditions. These values have been subsequently expressed in the form of the above-mentioned CHP maps—see Figure 5a–c. The solid contours marked by the labels correspond to the percentage values of the power dissipation efficiency (i.e., *η*, Equation (1)). The dashed contours then delimit the areas of assumed flow instability (i.e., the *ξ*-values ≤ 0, Equation (3)).

Generally, higher *η*-values are connected with promising thermomechanical conditions. As it was implied above, the *η*-values reflect the progress of metallurgical processes (most often the DRV or DRX softening) under various thermomechanical conditions. The progress of these softening processes, of course, corresponds also with the changes in the flow-stress level. Since the *η*-values are calculated form the flow-stress values, their mutual ability to reflect the dynamic softening progress should, thus, be evident. In the case of this research, for the purpose of easier comparison, the assembled CHP maps have been combined with the flow-stress (FS) maps by their mutual superimposing—see the color background expressing the flow stress evolution (Figure 5a–c).

It is well-known that the purpose of the *ξ*-values is to reveal potentially aggravated forming conditions. Nevertheless, as presented in the introduction, the calculated *ξ*-values do not have to be sufficient for the revealing of all these unstable conditions. To deal with this issue, Zhou et al. [1] introduced the above-mentioned activation-energy processing (AEP) maps. The assumption is as follows: Since the activation energy expresses the difficulty of the deformation course, the *Q*-values can be utilized to reveal potentially aggravated forming conditions and thus encourage the results given by the *ξ*-values. The AEP maps of the investigated steel, i.e., superimposition of the CHP maps (Section 2.3) over the AE maps (Section 2.4), are offered in Figure 5d–f. The contours embody the CHP maps—the same as in the case of Figure 5a–c—however, in this case, the color background corresponds with the evolution of activation energy. All the above-mentioned maps have been assembled by means of the Gnuplot 5.2 graphing utility Patchlevel 7 [60].

Based on the gained *η*-range, the deformation course of the studied material can be associated with the specific metallurgical processes, e.g., *η*-range from 30 to 50% is usually attributed to the DRX course, lower values correspond with the DRV and the *η*-values above the ca. 60% can have connection to the superplastic behavior [6,23,24]. In the case of the studied steel, the *η*-values go beyond the 30% threshold (see Figure 5)—Thus, the softening course is probably mediated via DRX. This is in accordance with the observations in Figure 3.

It is clear, the *η*-values increase as the temperature level increases. The obvious *η*-increase is also observable with the decrease of strain rate under medium temperature levels. This phenomenon can be observed also under higher temperatures—however, local *η*-maximums can be visible at the highest strain rates (see, e.g., the parabolic *η*-course under the 1468 K in Figure 5b). The *η*-increase is then practically negligible under lower temperatures. Nevertheless, despite of the nuances in *η*-$\dot{\varepsilon }$ dependence, it can be said that the *η*-values generally increase with increasing temperature and decreasing strain rate. This behavior is closely linked with the softening course. It is understandable that the higher the temperature and the lower the strain rate, the earlier the DRX-start. In other words, the *η*-values represent the intensity of the DRX progress—higher *η*-values are coupled with a more progressed DRX course.

**Figure 6:**
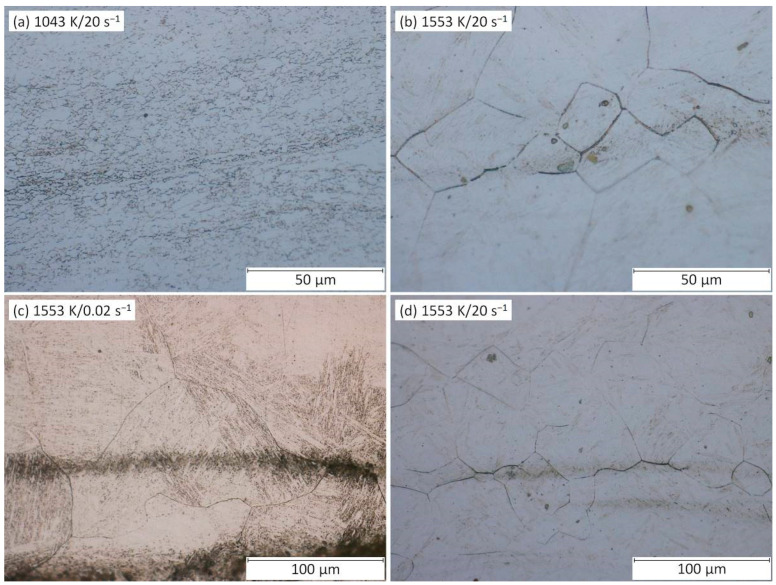
OM images of samples deformed under the true strain of 1.0. (**a**) 1043 K/20 s^−1^, (**b**) 1553 K/20 s^−1^, (**c**) 1553 K/0.02 s^−1^, (**d**) 1553 K/20 s^−1^.

The relation between the *η*-values and DRX course can be observed in optical microscopy (OM) images (Figure 6). Based on the comparison of Figure 6a,b, it can be seen that microstructure under the higher temperature is fully recrystallized (see clearly non-elongated grains with distinctly equiaxed boundaries), which is linked with a higher *η*-level in Figure 5c,f. Further, Figure 6c,d demonstrates the growing of recrystallized grains with the decrease in strain rate as the consequence of longer grain-growth time, which is also linked with a higher *η*-level. Of course, the grain growth is also supported by higher temperature levels.

- Thus, it can be stated, the more progressed the DRX and the larger the grains, the higher the *η*-level.
- It is understandable that a larger grain size is not beneficial for the achievement of simultaneous high strength and high toughness [9,16]. Thus, it seems, too much high *η*-level does not have to be connected with the best thermomechanical conditions—at least as regards to the final material properties. The growing grain size with the increasing *η*-level has been also observed, e.g., in [1,9].

The color background in Figure 5a–c distinctly illustrates the relation between the *η*-values and *σ*-values. It is observable, the *η*-values have practically an opposite evolution—they are rising as the *σ*-values are decreasing (i.e., inverse proportion). Thus, in connection to the flow stress course, higher *η*-values refer to deformation conditions which are beneficial form the point of view of lower forming forces and thus lower energetic consumption and also longer tool life.

The relation between the *η*-values and *Q*-values (see Figure 5d–f) is, however, quite different in comparison to the *η*-*σ* course. It is observable, if the temperature is rising, the *η*-values are increasing and *Q*-values are decreasing (i.e., the inverse proportion). Nevertheless, if the strain rate is rising, the *η*-values and *Q*-values are both decreasing (i.e., direct proportion). Similar *Q*-evolution has also been observed in [56].

- Note, the decrease of the *Q*-values is coupled with the easier deformation course. Specifically, the decrease of the *Q*-values with the increasing temperature is linked with the higher kinetic energy of dislocation movement. The decrease of the *Q*-values with the increasing strain rate is then linked with the increasing shear stress, i.e., with the activation of dislocation movement. A more detailed explanation can be found in [55,56].

It is discernable, the influence of the strain rate on the *Q*-values is rather less apparent—linked mainly with lower temperature levels. The strain rate influence is the most apparent under the temperature level of 1043 K at the true strain of 0.4 (Figure 5d). The *Q*-value decreased from 776 to 708 kJ·mol^−1^ when the strain rate increased from 0.02 to 20 s^−1^. With respect to the highest temperature level, i.e., 1553 K, the *Q*-value decreased from 255 to 233 kJ·mol^−1^ as the strain rate increased from 0.02 to 20 s^−1^.

- It is noticeable that this *Q*-decrease (representing a better deformation course) is in accordance with the decrease of the grain size due to the increasing strain rate as illustrated in Figure 6c,d.
- The raising of *η*-values and lowering of *Q*-values are commonly linked with the achieving of beneficial thermomechanical conditions. However, as stated above, the generally beneficial increase of *η*-values is in the same time coupled with the grain size growing (i.e., with the reaching of worse strength-toughness combination) because the *η*-increase is closely linked with an increasing temperature and decreasing strain rate. On the other hand, the generally beneficial decrease of *Q*-values is linked with a grain size growing only at the temperature increase.
- Nonetheless, the strain rate effect is quite opposite when the true strain achieves the highest level, i.e., 1.0 (Figure 5f). At the same temperature (specifically 1043 K), the *Q*-value increased from 699 to 725 kJ·mol^−1^ as the strain rate increased from 0.02 to 20 s^−1^. At the highest temperature level (i.e., 1553 K), the *Q*-value increased from 263 to 273 kJ·mol^−1^ as the strain rate increased from 0.02 to 20 s^−1^. Thus, it seems like that under the higher strains the *Q*-evolution and *η*-evolution become to be inverse proportional even in the case of the strain rate course.

**Figure 7:**
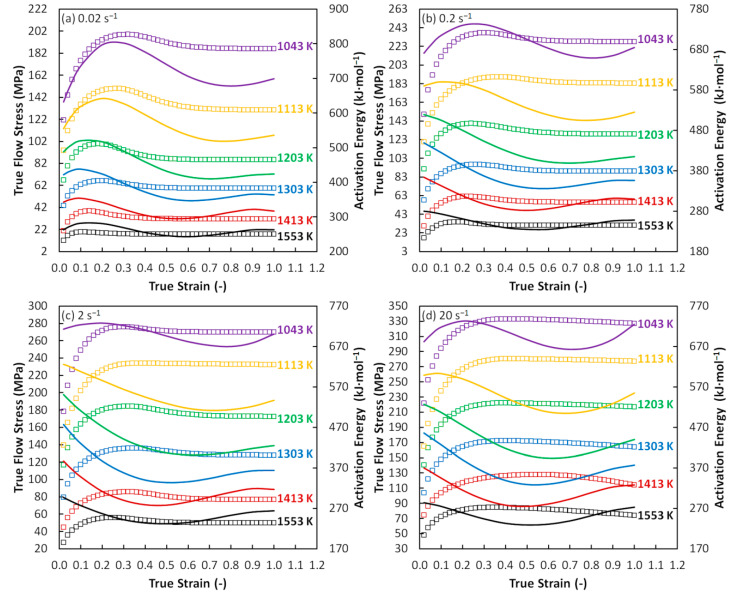
Correlation between the activation energy and flow stress course. (**a**) Strain rate of 0.02 s^−1^; (**b**) strain rate of 0.2 s^−1^; (**c**) strain rate of 2 s^−1^; (**d**) strain rate of 20 s^−1^. Boxes–experimental flow curves; solid lines–activation energy evolution.

Furthermore, Figure 7 offers a detailed view on the relationship between the calculated values of activation energy (solid lines) and strain level. This relation is in the same time compared with the flow stress course (boxes).

It is visible that the stage of the *σ*-increase is under a strain rate of 0.02 s^−1^ coupled with the increase of *Q* (see e.g., 1043 K/0.02 s^−1^). This *Q*-course seems to be related to the prevailing work hardening (i.e., aggravated deformation conditions). The observed *Q*-increase is then terminated by the achieving of maximum point with a following gradual decrease. This decrease can be then considered as the manifestation of the prevailing DRX course (also manifested by the *σ*-decrease) which is associated with better deformation conditions. Nevertheless, most of the *Q*-*ε* curves start immediately with a decrease phase regardless to the flow curve peak point. In addition, the decrease in *Q*-values is not associated with the following constant phase (equilibrium between the DRX and work hardening) as is typical for the *σ*-*ε* curves. Moreover, the following *Q*-increase with strain can be observed in the case of all *Q*-*ε* curves. This increase is under higher temperatures and lower strain rates followed by another decrease. The observed increase signalizes other aggravation of deformation conditions although the corresponding *σ*-ε curves remain in constant phase. This fact can result in divergences in the prediction of unfavorable conditions via the above-discussed CHP and AEP maps.

**Figure 8:**
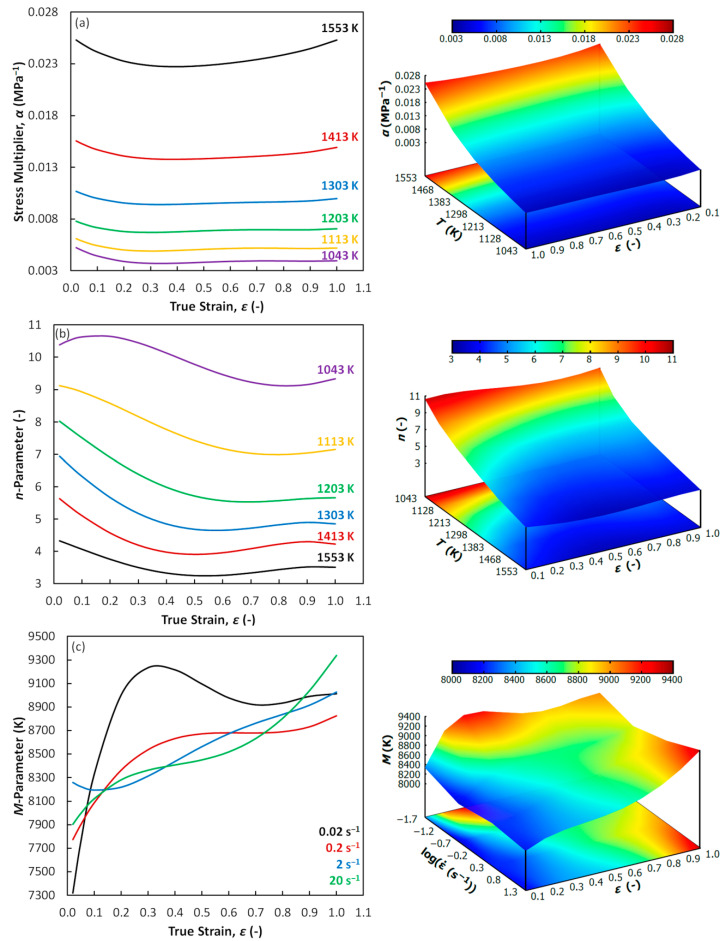
Evolution of the parameters for the activation energy calculation. (**a**) Stress multiplier *α*; (**b**) parameter *n*; (**c**) parameter *M*.

In addition, Figure 8 shows the evolution of the parameters which were calculated as an intermediate step for the activation energy maps assembling.

Figure 8a is aimed on the stress multiplier *α*. It is clear that this parameter is predominantly influenced by the temperature level and almost independent on the strain course. At first glance it is clear that the temperature dependence of the *α*-parameter is opposite in comparison to the temperature dependence of *Q*-values (Figure 7). However, the differences in the *α*-values between the highest and the lowest temperature levels are almost negligible. With respect to the *n*-parameter (see Figure 8b), the temperature and strain dependencies are very similar to those observed in *Q*-evolution (Figure 7). The course of the *M*-parameter (Figure 8c) is then more complicated. It can be seen that the strain dependency is, in the case of this parameter, strongly influenced by the strain rate level. Small strain rate values (0.02 and 0.2 s^−1^) are associated with the most complicated course while the course under the higher rates seems to be simpler. It seems that this behavior can strongly influence the reaction of the *Q*-values on the changes in strain rate level (consider Equation (4)). As observed in Figure 5d, the *Q*-values are higher under the lower strain rates at the strain of 0.4, which corresponds with the observation under the strain of 0.4 in Figure 8c. The situation is opposite in the case of the strain of 1.0 (Figure 5f). This is also reflected by the *M*-course in Figure 8c—the *M*-values of the rates of 2 and 20 s^−1^ become to be higher. In addition, the complicated *M*-course is also manifested by the more complicated form of the polynomial description—compare the values in Table 3 (*M*-polynomial) with the values in Table 4 (*n*-polynomial) and Table 5 (*α*-polynomial). Table 3 does not contain zero values unlike the other tables, i.e., the full polynomial form had to be applied to properly describe the *M*-course. The complicated *M*-evolution is also apparent from the surface expression in Figure 8.

**Figure 9:**
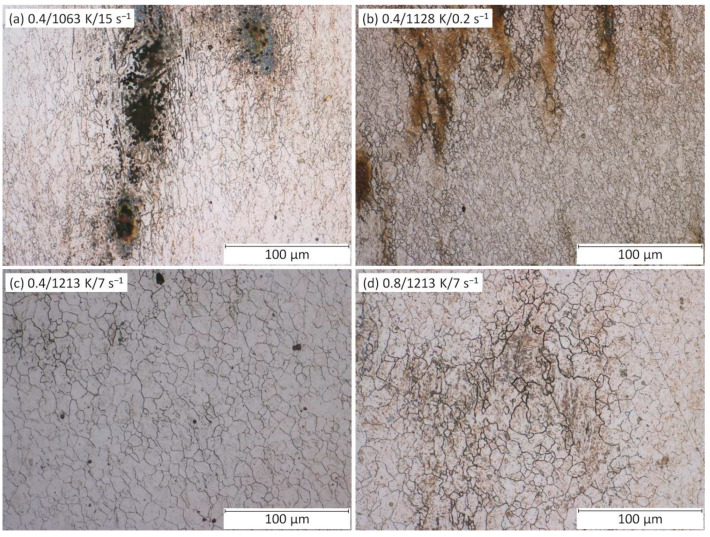
OM images of samples deformed under various strains. (**a**) 0.4/1063 K/15 s^−1^; (**b**) 0.4/1128 K/0.2 s^−1^; (**c**) 0.4/1213 K/7 s^−1^; (**d**) 0.8/1213 K/7 s^−1^.

Furthermore, the flow instability areas (i.e., *ξ*-values ≤ 0) bring the information about the potential presence of metallurgical instabilities which can accompany the deformation course. Based on the formed material and thermomechanical circumstances, these instability areas can be appeared as the manifestation of e.g., flow localization, shear bands, Lüders’ bands, kink bands, mechanical twinning, or cracks [1,6]. In the case of the above presented maps (Figure 5), two areas of metallurgical instability can be observed:

- As regards to the strain of 0.4, the first instability area (I) is situated in the very small temperature range of 1043–ca. 1128 K and the strain rate range of 0.02–ca. 0.2 s^−1^. It is observable, this small area is growing with the increase of strain—especially towards to higher strain rates. Under the strain of 1.0, the district (I) covers the temperature range of 1043–ca. 1170 K and the strain rate range of 0.02–20 s^−1^.
- Under the strain of 0.4, the second area (II) is located in the wide temperature range of ca. 1086–more than 1298 K and the wide strain rate range of ca. 0.06–20 s^−1^. This area, however, is under the higher strains significantly reduced.
- It is observable, as the strain level increase the instability district (II) gives way to district (I). Practically, both districts are probably the part of the same instability domain.
- It is noticeable, the location of both districts is in the accordance with the thermomechanical conditions which are connected with the higher values of activation energy, higher flow stress values and lower values of power dissipation efficiency, i.e., with conditions potentially associated with an aggravated deformation course.
- Nevertheless, neither the microstructure observation (realized inside of the instability districts and in a surrounding area) (Figure 9) nor the flow curve course (Figure 3) proves the apparent manifestation of the typical above-mentioned instability features. Only inclusions and segregations can be visible as the consequence of the effort to visualize original grain boundaries via etching procedure (Figure 9). It should be noted that under the constant temperature the *ξ*-values are sensitive to the changes in *σ*-value with the strain rate level. Unfortunately, these changes can be negatively influenced in the stage of data-acquiring procedure. In addition, the final processing-map form is influenced by the subsequent data processing, e.g., utilized surface-interpolation methods. These facts can lead to the overestimation of results and microstructural observations then should confirm or refute these results.

The results have showed that the activation energy maps can be used as a support tool for the choice of appropriate forming conditions in cooperation with the conventional processing maps. As stated previously in [1] the main benefit of the AE maps is that they consider the difficulty of the deformation course. The above discussed correlation issue can enrich the overall awareness regarding the processing maps theory and can lead to the selecting of more useful forming conditions.

## 4. Conclusions

For the case of Cr-Mo low-alloyed steel, based on Prasad’s dynamic material model, conventional hot processing maps, i.e., the maps of power dissipation efficiency (*η*) combined with the maps of metallurgical instability (*ξ*), have been assembled and subsequently superimposed over the maps of flow stress (*σ*) evolution and also over the maps of activation energy (*Q*) evolution.

Two flow curve datasets have been combined to assemble the mentioned maps. The experimental one has been acquired via a series of uniaxial hot compression tests realized up to the true strain of 1.0 in the temperature range of 1043–1553 K and the strain rate range of 0.02–20 s^−1^. In order to gain a higher number of data points inside the experimental matrix, a multi-layer perceptron network has been assembled, trained, and subsequently utilized to predict the flow stress course under five additional temperature levels.

Based on the assembled maps, a correlation among the power dissipation efficiency, flow stress course, and activation energy evolution of the investigated steel has been studied. The basic presumption is: Higher *η*-values, lower *Q*-values, and lower *σ*-values should be connected with beneficial thermomechanical conditions. Correlation among these indicators should be natural since the relation between the flow stress and strain rate is utilized to calculate the *η* and *Q* values.

The *η*-evolution and *σ*-course are inversely proportional (the *η*-increase is connected with the *σ*-decrease). This indicates that *η*-increase is connected with the achieving of lower forming forces and energy consumption, and also with the progress of the softening course.

The *η*-evolution and *Q*-evolution are then proportional inversely with regard to the change in a temperature level (*η*-values increase as *Q*-values decrease). This indicates that *Q*-evolution and also *η*-evolution predict more beneficial conditions under the higher temperature levels. However, except of higher strains, they are non-unified with respect to the strain rate (both decrease as the strain rate increase). Thus, in contrast with the *η*-values, the *Q*-values predict better conditions under higher strain rates. Note, higher strain rates are beneficial with respect to the smaller grain size (providing a better strength-toughness combination).

Furthermore, the assembled processing maps reveal two instability districts. Both districts have been observed under the lower temperature levels, lower *η*-values, higher *Q*-values, and higher *σ*-values, i.e., under conditions linked with a harder deformation course.

The obtained findings can contribute to the enrichment of overall awareness about the processing maps theory, which can lead to the selecting of better forming conditions.

## Figures and Tables

**Table 1:** Chemical composition of the investigated Cr-Mo low-alloyed steel in wt%.

C	Cr	Mo	Mn	Si	Al	N
0.29	0.79	0.21	1.20	0.27	0.028	0.0093

**Table 2:** Distribution of the temperature and strain rate combinations for the MLP learning purpose.

$\dot{\varepsilon }$ (s^−1^)/*T* (K)	1553	1413	1303	1203	1113	1043
**0.02**	train	test	valid	train	test	train
**0.2**	valid	train	train	test	valid	train
**2**	train	valid	test	train	train	test
**20**	test	train	train	valid	train	valid

**Table 3:** Material constants *a_ij_* of the polynomial *M*($\varepsilon ,\;\dot{\varepsilon }$) (7).

*a_ij_*	Value	*a_ij_*	Value	*a_ij_*	Value	*a_ij_*	Value	*a_ij_*	Value
*a*_0,0_	8.95 × 10^3^	*a*_1,0_	4.73 × 10^3^	*a*_2,0_	−3.88 × 10^3^	*a*_3,0_	−8.05 × 10^3^	*a*_4,0_	1.02 × 10^4^
*a*_0,1_	−4.75 × 10^2^	*a*_1,1_	−7.10 × 10^3^	*a*_2,1_	1.50 × 10^4^	*a*_3,1_	−6.14 × 10^3^	*a*_4,1_	−3.86 × 10^3^
*a*_0,2_	−7.86 × 10^2^	*a*_1,2_	−4.11 × 10^3^	*a*_2,2_	7.21 × 10^3^	*a*_3,2_	1.28 × 10^3^	*a*_4,2_	−6.33 × 10^3^
*a*_0,3_	9.56 × 10^1^	*a*_1,3_	8.54 × 10^2^	*a*_2,3_	−1.73 × 10^3^	*a*_3,3_	4.16 × 10^2^	*a*_4,3_	7.78 × 10^2^
*a*_0,4_	5.94 × 10^1^	*a*_1,4_	4.24 × 10^2^	*a*_2,4_	−8.50 × 10^2^	*a*_3,4_	1.72 × 10^2^	*a*_4,4_	4.25 × 10^2^
*R*	0.996611								

**Table 4:** Material constants *b_ij_* of the polynomial *n*(*ε*,*T*) (8).

*b_ij_*	Value	*b_ij_*	Value	*b_ij_*	Value	*b_ij_*	Value	*b_ij_*	Value
*b*_0,0_	5.67 × 10^2^	*b*_1,0_	6.53 × 10^2^	*b*_2,0_	−7.35 × 10^2^	*b*_3,0_	2.75 × 10^2^	*b*_4,0_	−6.53 × 10^0^
*b*_0,1_	−1.71 × 10^0^	*b*_1,1_	−1.33 × 10^0^	*b*_2,1_	1.30 × 10^0^	*b*_3,1_	−3.87 × 10^−1^	*b*_4,1_	0.00 × 10^0^
*b*_0,2_	1.97 × 10^−3^	*b*_1,2_	8.70 × 10^−4^	*b*_2,2_	−7.04 × 10^−4^	*b*_3,2_	1.40 × 10^−4^	*b*_4,2_	0.00 × 10^0^
*b*_0,3_	−1.01 × 10^−6^	*b*_1,3_	−1.85 × 10^−7^	*b*_2,3_	1.12 × 10^−7^	*b*_3,3_	0.00 × 10^0^	*b*_4,3_	0.00 × 10^0^
*b*_0,4_	1.93 × 10^−10^	*b*_1,4_	0.00 × 10^0^	*b*_2,4_	0.00 × 10^0^	*b*_3,4_	0.00 × 10^0^	*b*_4,4_	0.00 × 10^0^
*R*	0.996660								

**Table 5:** Material constants *c_ij_* of the polynomial *α*(*ε*,*T*) (9).

*c_ij_*	Value	*c_ij_*	Value	*c_ij_*	Value	*c_ij_*	Value	*c_ij_*	Value
*c*_0,0_	2.85 × 10^−2^	*c*_1,0_	−2.84 × 10^−1^	*c*_2,0_	2.86 × 10^−1^	*c*_3,0_	−1.06 × 10^−1^	*c*_4,0_	1.58 × 10^−2^
*c*_0,1_	−8.81 × 10^−5^	*c*_1,1_	5.65 × 10^−4^	*c*_2,1_	−4.64 × 10^−4^	*c*_3,1_	1.02 × 10^−4^	*c*_4,1_	0.00 × 10^0^
*c*_0,2_	1.32 × 10^−7^	*c*_1,2_	−3.74 × 10^−7^	*c*_2,2_	2.57 × 10^−7^	*c*_3,2_	−3.82 × 10^−8^	*c*_4,2_	0.00 × 10^0^
*c*_0,3_	−9.99 × 10^−11^	*c*_1,3_	7.75 × 10^−11^	*c*_2,3_	−3.76 × 10^−11^	*c*_3,3_	0.00 × 10^0^	*c*_4,3_	0.00 × 10^0^
*c*_0,4_	3.26 × 10^−14^	*c*_1,4_	0.00 × 10^0^	*c*_2,4_	0.00 × 10^0^	*c*_3,4_	0.00 × 10^0^	*c*_4,4_	0.00 × 10^0^
*R*	0.999562								

## References

[1] Zhou P., Deng L., Zhang M., Gong P., Wang X.-Y. (2019). Characterization of hot workability of 5052 aluminum alloy based on activation energy-processing map. J. Mater. Eng. Perform..

[2] Qian P., Tang Z., Wang L., Siyasiya C.W. (2020). Hot Deformation characteristics and 3-D processing map of a high-titanium Nb-micro-alloyed steel. Materials.

[3] Liu D., Ding H., Cai M., Han D. (2019). Hot Deformation behavior and processing map of a Fe-11Mn-10Al-0.9C duplex low-density steel susceptible to κ-carbides. J. Mater. Eng. Perform..

[4] Quan G.Z., Zhao L., Chen T., Wang Y., Mao Y.P., Lv W.Q., Zhou J. (2012). Identification for the optimal working parameters of as-extruded 42CrMo high-strength steel from a large range of strain, strain rate and temperature. Mater. Sci. Eng. A.

[5] Xu L., Chen L., Chen G., Wang M. (2018). Hot deformation behavior and microstructure analysis of 25Cr3Mo3NiNb steel during hot compression tests. Vacuum.

[6] Opěla P., Kawulok P., Kawulok R., Kotásek O., Buček P., Ondrejkovič K. (2019). Extension of experimentally assembled processing maps of 10CrMo9-10 steel via a predicted dataset and the influence on overall informative possibilities. Metals.

[7] Zhang C., Zhang L., Shen W., Liu C., Xia Y., Li R. (2016). Study on constitutive modeling and processing maps for hot deformation of medium carbon Cr–Ni–Mo alloyed steel. Mater. Des..

[8] Kliber J., Schindler I., Kawulok P., Sedláček R. (2018). Energy dissipation and instability parameter at high temperature forming of middle carbon steel. Proceedings of the 26th International Conference on Metallurgy and Materials.

[9] Yang Z., Li Y., Li Y., Zhang F., Zhang M. (2016). Constitutive modeling for flow behavior of medium-carbon bainitic steel and its processing maps. J. Mater. Eng. Perform..

[10] Kumar N., Kumar S., Rajput S.K., Nath S.K. (2017). Modelling of flow stress and prediction of workability by processing map for hot compression of 43CrNi steel. ISIJ Int..

[11] Gao X.J., Jiang Z.Y., Wei D.B., Jiao S.H., Chen D.F. (2014). Study on hot-working behavior of high carbon steel/low carbon steel composite material using processing map. Key Eng. Mater..

[12] Zhou Y., Liu Y., Zhou X., Liu C. (2015). Processing maps and microstructural evolution of the type 347H austenitic heat-resistant stainless steel. J. Mater. Res..

[13] Zhang P., Hu C., Ding C.-G., Zhu Q., Qin H.-Y. (2015). Plastic deformation behavior and processing maps of a Ni-based superalloy. Mater. Des..

[14] Mingjie Z., Fuguo L., Shuyun W., Chenyi L. (2010). Characterization of hot deformation behavior of a P/M Nickel-base superalloy using processing map and activation energy. Mater. Sci. Eng. A.

[15] Srinivasan N., Prasad Y.V.R.K. (1994). Microstructural control in hot working of IN-718 superalloy using processing map. Metall. Mater. Trans. A..

[16] Wang Y., Jiang S., Zhang Y. (2017). Processing map of NiTiNb shape memory alloy subjected to plastic deformation at high temperatures. Metals.

[17] Quan G.-Z., Zou Z.-Y., Wang T., Liu B., Li J.-C. (2017). Modeling the hot deformation behaviors of as-extruded 7075 aluminum alloy by an artificial neural network with back-propagation algorithm. High. Temp. Mater. Process..

[18] Liu R., Wang W., Chen H., Zhang Y., Wan S. (2019). Hot deformation and processing maps of B_4_C/6061Al nanocomposites fabricated by spark plasma sintering. J. Mater. Eng. Perform..

[19] Zhang J., Di H., Wang H., Mao K., Ma T., Cao Y. (2012). Hot deformation behavior of Ti-15-3 titanium alloy: A study using processing maps, activation energy map, and Zener–Hollomon parameter map. J. Mater. Sci..

[20] Prasad Y.V.R.K., Gegel H.L., Doraivelu S.M., Malas J.C., Morgan J.T., Lark K.A., Barker D.R. (1984). Modeling of dynamic materials behavior in hot deformation: Forging of Ti-6242. Metall. Trans. A.

[21] Meng Q., Bai C., Xu D. (2018). Flow behavior and processing map for hot deformation of ATI425 titanium alloy. J. Mater. Sci. Technol..

[22] Zhang S., Liang Y., Xia Q., Ou M. (2019). Study on tensile deformation behavior of TC21 titanium alloy. J. Mater. Eng. Perform..

[23] Chakravartty J.K., Prasad Y.V.R.K., Asundi M.K. (1991). Processing map for hot working of Alpha-Zirconium. Metall. Trans. A.

[24] Saxena K.K., Yadav S.D., Sonkar S., Pancholi V., Chaudhari G.P., Srivastava D., Dey G.K., Jha S.K., Saibaba N. (2014). Effect of temperature and strain rate on deformation behavior of Zirconium alloy: Zr-2.5Nb. Procedia Mater. Sci..

[25] Suresh K., Dharmendra C., Rao K.P., Prasad Y.V.R.K., Gupta M. (2015). Processing map of AZ31-1Ca-1.5 vol% nano-alumina composite for hot working. Mater. Manuf. Process..

[26] Zhang Y., Sun H., Volinsky A.A., Tian B., Song K., Chai Z., Liu P., Liu Y. (2016). Dynamic recrystallization behavior and processing map of the Cu–Cr–Zr–Nd alloy. Springer Plus.

[27] Duan Y., Ma L., Qi H., Li R., Li P. (2017). Developed constitutive models, processing maps and microstructural evolution of Pb-Mg-10Al-0.5B alloy. Mater. Charact..

[28] Łyszkowski R., Bystrzycki J. (2006). Hot deformation and processing maps of an Fe_3_Al intermetallic alloy. Intermetallics.

[29] Wang S., Luo J.R., Hou L.G., Zhang J.S., Zhuang L.Z. (2017). Identification of the threshold stress and true activation energy for characterizing the deformation mechanisms during hot working. Mater. Des..

[30] GLEEBLE: Gleeble^®^Thermal-Mechanical Simulators. https://gleeble.com/.

[31] Opěla P., Schindler I., Kawulok P., Kawulok R., Rusz S., Rodak K. (2019). Hot flow curve description of CuFe2 alloy via different artificial neural network approaches. J. Mater. Eng. Perform..

[32] Opěla P., Schindler I., Rusz S., Navrátil H. (2019). A hot flow curve approximation via biology-inspired algorithms. Proceedings of the 28th International Conference on Metallurgy and Materials.

[33] Opěla P., Schindler I., Očenášek V., Kawulok P., Kawulok R., Rusz S. (2019). Modelling the hot deformation behavior of AlSi1MgMn alloy via flow stress models utilizing intelligent algorithms. Proced. Struct. Integr..

[34] McCulloch W.S., Pitts W.H. (1943). A logical calculus of ideas immanent in nervous activity. Bull. Math. Biophys..

[35] Rosenblatt F. (1958). The perceptron: A probabilistic model for information storage and organization in the brain. Psychol. Rev..

[36] Krenker A., Bešter J., Kos A., Suzuki K. (2011). Introduction to the artificial neural networks. Artificial Neural Networks Methodological Advances and Biomedical Applications.

[37] Debes K., Koenig A., Gross H.M. Transfer Functions in Artificial Neural Networks: A Simulation-based Tutorial. https://www.brains-minds-media.org/archive/151/.

[38] Gauss J.C.F. (1823). Theory of the Combination of Observations Least Subject to Errors.

[39] Levenberg K. (1944). A method for the solution of certain non-linear problems in least squares. Quart. Appl. Math..

[40] Marquardt D.W. (1963). An algorithm for least-squares estimation of nonlinear parameters. J. Soc. Indust. Appl. Math..

[41] Roweis S. Levenberg-marquardt Optimization. https://cs.nyu.edu/~roweis/notes/lm.pdf.

[42] Bayes T., Price R. (1763). An essay towards solving a problem in the doctrine of chance. By the late Rev. Mr. Bayes, F.R.S. communicated by Mr. Price, in a letter to John Canton, A.M.F.R.S. Phil. Trans..

[43] MacKey D.J.C. (1992). Bayesian interpolation. Neural Comput..

[44] Rumelhart D.E., Hinton G.E., Williams R.J., Feldman J.A., Hayes P.J., Rumelhart D.E. (1986). Learning internal representations by error propagation. Parallel Distributed Processing: Explorations in the Microstructure of Cognition.

[45] MathWorks MATLAB^®^ Math. Graphics. Programming. https://www.mathworks.com/products/matlab.html.

[46] Beale M.H., Hagan M.T., Demuth H.B. Neural Network Toolbox^TM^ 7: User’s Guide. https://www2.cs.siu.edu/~rahimi/cs437/slides/nnet.pdf.

[47] Alexander J.M., Lenard J.G. (1989). Mapping dynamic material behaviour. Modelling Hot Deformation of Steels.

[48] Gegel H.L., Malas J.C., Doraivelu S.M., Shende V.A., Semiatin S.L. (1996). Modeling techniques used in forging process design: Dynamic material modeling. ASM Handbook.

[49] Kumar A.K.S.K. (1987). Criteria for predicting metallurgical instabilities in processing. Master’s Thesis.

[50] Prasad Y.V.R.K. (1990). Recent Advances in the Science of Mechanical processing. Indian J. Technol..

[51] Schindler I., Kawulok P., Kawulok R., Hadasik E., Kuc D. (2013). Influence of calculation method on value of activation energy in hot forming. High Temp. Mater. Process..

[52] Schindler I., Kawulok P., Hadasik E., Kuc D. (2013). Activation energy in hot forming and recrystallization models for magnesium alloy AZ31. J. Mater. Eng. Perform..

[53] Kawulok P., Schindler I., Kawulok R., Opěla P., Sedláček R. (2018). Influence of heating parameters on flow stress curves of low-alloy Mn-Ti-B steel. Arch. Metall. Mater..

[54] Schindler I., Kawulok P., Očenášek V., Opěla P., Kawulok R., Rusz S. (2019). Flow stress and hot deformation activation energy of 6082 aluminium alloy influenced by initial structural state. Metals.

[55] Mohamadizadeh A., Zarei-Hanzaki A., Abedi H.R. (2016). Modified constitutive analysis and activation energy evolution of a low-density steel considering the effects of deformation parameters. Mech. Mater..

[56] Liu L., Wu Y.X., Gong H., Wang K. (2019). Modification of constitutive model and evolution of activation energy on 2219 aluminum alloy during warm deformation process. Trans. Nonferrous Met. Soc. China.

[57] Garofalo F. (1963). An empirical relation defining the stress dependence of minimum creep rate in metals. Trans. Metall. Soc AIME.

[58] Simpson T. (1755). A letter to the Right Honourable George Earl of Macclesfield, President of the Royal Society, On the Advantage of Taking the Mean of a Number of Observations in Practical Astronomy. Philos. Trans..

[59] Pearson K. (1895). Note on regression and inheritance in the case of two parents. Proc. R. Soc. Lond..

[60] Gnuplot: Portable Command-line Driven Graphing Utility. http://www.gnuplot.info/.

